# Update burn surgery: overview of current multidisciplinary treatment concepts

**DOI:** 10.1515/iss-2024-0020

**Published:** 2024-08-23

**Authors:** Frederik Schlottmann, Lisa Lorbeer

**Affiliations:** Department of Plastic, Aesthetic, Hand and Reconstructive Surgery, Hannover Medical School, Hannover, Germany

**Keywords:** burns, burn units, intensive care units, plastic surgery procedures, rehabilitation, skin transplantation

## Abstract

The treatment of severe burn injuries is an essential part of plastic-reconstructive surgery. Severe burned patients are treated in burn centers, which have highly specialized technical and personnel equipment. In addition to clear recommendations for prehospital management, intensive care therapy is usually required for extensive burn wounds. Shock therapy in burns primarily involves balanced fluid resuscitation according to hemodynamic monitoring, vasopressor support, pain management, temperature regulation, oxygen therapy, and comprehensive supportive care to stabilize the patient’s condition. Surgical treatment is still based on wound debridement and the gold standard of autologous split-thickness skin grafting. Besides skin transplantation, surgical management of burns may also involve the application of various topical therapies to promote wound healing, reduce pain, and prevent infection. These therapies may include antimicrobial dressings, skin substitutes, growth factors, or specialized wound care products. Once the acute treatment has been completed, multidisciplinary rehabilitation treatment takes place, which begins in the acute hospital and continues in the outpatient and inpatient rehabilitation areas. Surgical treatment of the secondary complications of burns and scars is also an important component of burn care. Comprehensive knowledge of the various components and players involved in the care of severely burned patients is, therefore, required in order to achieve the best possible outcome for the patient.

## Introduction

The history of wound treatment and skin grafts for burn injuries goes back centuries. Nowadays, specialized burn care and treatment is available to optimize patient outcomes [1].

According to surveys by the German Society for Burn Medicine (DGV), a total of 4,350 people in Germany were affected by burn injuries in 2016 that led to treatment in a burn intensive care unit (ICU) [2]. A significantly higher number of burn injuries that were not treated in burn units can be assumed. Most burns occur in adults (up to 70 %) and children (up to 95 %) in household and leisure activities [3]. A large number of burn accidents are preventable. Prevention programs such as the use of smoke alarms in private homes have led to a significant reduction in the incidence of burn accidents in industrial countries. In Germany, mandatory smoke alarms in private homes were implemented in 2016. Other burn prevention strategies are, e.g., standard operation procedure protocols in work facilities and flameproof shielding gear. Burn prevention strategy programs have also demonstrated cost effectiveness [4]. In the 21st century, social media is a possible modality to raise public awareness of burn accident prevention [5].

The onset of the coronavirus pandemic in 2020 had a significant impact on society and the healthcare system. Consequently, there was a reduction in the total number of operations and the elective surgery program. Even COVID has had an impact on burns victims. Interestingly, in comparison to the early (2020) and late (2021) phases, more burn patients were discharged from the emergency department to the outpatient setting just before the COVID pandemic (in 2019) [6]. Overall, there was an increase in inpatient interventions at the onset of the COVID pandemic [6]. However, the change in surgical procedure was also observed after the increase in the elective program.

Burn ICUs are equipped with advanced medical technology and staffed with highly trained professionals, such as burn surgeons, nurses specialized in burn care, physiotherapists, and psychologists to provide optimal burn care throughout Germany [7]. Admission to burn ICUs is typically based on the severity of the burn injury, the need for specialized care, medical stability, and consideration of individual patient factors. Following criteria should lead to referral to a burn unit: 2° burns of 10 % and more body surface area, 3° burns, burns to hands, face and genital, burns caused by electricity including lightning, chemical burns and inhalatory trauma, and burn patients with concomitant diseases that complicate treatment. Treatment in a burn center should be considered for burn patients with special needs for psychological, psychiatric, or physical care [3]. Admission may occur following referral from emergency departments, trauma centers, or other healthcare facilities. Consultation with burn specialists or multidisciplinary teams may help determine the appropriateness of admission and guide the initial management plan. Special consideration may be given to pediatric or elderly patients with burns due to their increased vulnerability and unique care needs. Pediatric burn units may offer specialized services and a child-friendly environment to accommodate the needs of young patients. The treatment costs of severely burned patients in acute care differs between countries and also depend on the patient’s age, the burn mechanism, the depth of the burn, and the TBSA [8]. The average costs are higher with increasing age, more severe burns, inhalation injury, electrical burns, and in nonsurvivors. Therefore, the average daily cost of burn care during hospital stay varies and ranges from 3,275.9 USD to 3,677.35 USD [8].

Typical causes of burns recorded in a burn center in 2022 are in descending order of frequency, flames (including deflagration 44 %), scalds (21 %), fat burns (including oil, 3 %), blistering skin diseases (3 %), explosion (2 %), electricity, acid (1 %), and alkali (1 %) [3]. The multidisciplinary treatment approach focuses on immediate stabilization of patients, pain management, and wound care to prevent complications such as infections and organ failure [7]. Involving specialists from various medical fields, burn units aim to regain function and mobility, as well as psychological well-being after sustaining burn. Overall, burn units in Germany are dedicated to providing high-quality specialized care for burn patients, aiming to improve outcomes and enhance their quality of life. The individual components of the multimodal treatment approach will be explained in detail below.

## Methods

A PubMed-based literature search was conducted using the terms “burn care standards” and “burn care guidelines.” Additionally, the current guideline recommendations from the Deutsche Gesellschaft für Verbrennungsmedizin and the American Burn Association were examined for further relevant literature. The search focused primarily on reviews and systematic reviews published between 2018 and 2024. Out of 935 identified research items, 285 were selected for further analysis after abstract review. Ultimately, 77 research items were included in the preparation of this manuscript.

### Initial care and preclinical management

Regardless of the accident mechanism, it is essential for the emergency services to assess the scene quickly and effectively in order to initiate the necessary measures. A risk analysis with prioritization of self-protection and standardized procedures as advised in the Advanced Trauma Life Support (ATLS) and Advanced Cardiac Life Support (ACLS) are recommended to optimize patient outcomes and minimize complications associated with burn injuries [9]. The preclinical management of burns refers to the immediate actions taken at the scene of a burn injury before the patient reaches a medical facility. This phase is critical for minimizing further damage, stabilizing the patient, and preparing them for definitive medical care. Effective preclinical management of burns requires prompt assessment, appropriate interventions, and coordination among first responders. The treatment of burned patients should also be based on established schemes such as the ABCDE scheme. First of all, it must be ensured that the airway is clear; if it is not clear or secured, intubation must be considered. Classic criteria such as respiratory insufficiency, lack of protective reflexes, and risk of airway obstruction are obvious indications for intubation [9]. The criteria of the American Burn Association (ABA) and the Denver criteria are also used as a guideline for early intubation [10]. The Denver criteria supplement those of the American Burn Association by two aspects: singed facial or nasal hair and suspected smoke inhalation. Intubation should be critically considered on the basis of the criteria in order to reduce unnecessary intubation and the associated risks. Complications related to intubation and ventilation occurred in 40 % of intubated patients who did not fulfill any or one of the intubation criteria [11].

Hypothermia prophylaxis is an important measure, because it represents a prognostically negative factor for the overall survival of burn victims [12]. Thermal monitoring begins preclinically and active warming is recommended at the start of the emergency phase, especially for patients with thermomechanical combination injuries [13]. On the contrary, active cooling must be stopped to minimize the risk of hypothermia [13]. Sterile dressings and warming foils are useful for hypothermia prophylaxis. Moreover, sterile dressings can also minimize pain by reducing draught [14]. No standardized concept for preclinical analgesia exists, but it should be given intravenously. Fluid over-resuscitation should be avoided preclinically; therefore, it is recommended to start with 1,000 mL of balanced crystalloid infusion therapy in adults within the first 2 h. Prewarmed infusions are also useful as hypothermia prophylaxis. The Parkland-Baxter formula and other known regimens are not recommended for fluid substitution in the preclinical setting [15].

In Germany, a central contact point run by the fire brigade in Hamburg is responsible for coordinating bed capacities for severely burned patients on a national level [14]. Communication and documentation are crucial for a smooth process. Early contact with the burn center enables efficient preparation. Suspected concomitant injuries should already be mentioned here to ensure that other specialist disciplines can be consulted in advance for a joint assessment in the trauma room. In addition, primary care in a nearby hospital can be useful in some cases. The transfer to a burn center occurs after the patient has stabilized.

### Intensive care treatment in burn units

The prognosis of a burn patient mainly depends on the parameters burned total body surface area (TBSA) and depth of burns, concomitant injuries, preexisting comorbidities, and the quality of medical care. A burn ICU is usually run in partnership by a plastic surgeon and an anesthesiologist [7]. There are currently a variety of scores to provide a subjective prognosis assessment of mortality and outcome on admission. The Abbreviated Burn Severity Index (ABSI) in particular has been proven to be a valid tool for prognosis prediction and has also been validated at national level in Germany [16]. The ABSI takes into account the parameters of gender, age, percentage of TBSA burned, inhalation trauma, and 3° burns using a point system of 2–13 points, from which a probability of survival can be calculated. The burn patient is presented to the burn unit team using standardized protocols analogous to polytrauma care. When possible, a detailed medical history should be taken, if necessary with the assistance of third parties, in order to obtain the best possible impression of the trauma mechanism. Regardless of the burn injury, the patient should undergo general trauma diagnostics according to trauma room protocols with the involvement of trauma surgery and broadly indicated CT diagnostics if the trauma mechanism is corresponding or unclear [17]. After securing the airway and establishing large-volume vascular access and invasive circulatory monitoring, the burn wounds are assessed surgically. A thorough assessment of the burn injury should be conducted to determine its severity, depth, and extent according to the above-mentioned guidelines. To assess the depth of the burn, the burn vesicles should be removed and the wounds cleaned of soot. Burned hair should be generously shaved before washing the entire patient with disinfectant solution. Hypothermia should be prevented by preheating the hydrotherapeutic care unit and working in a time-efficient manner [14]. Preclinical information regarding the extent and depth of the burn should be critically reviewed and verified by an experienced burn surgeon. When available, Laser Doppler Imaging technology should be used for further clarification of burns of undetermined depth [18]. For forensic and documentation reasons, photographic documentation should be taken [14]. Inhalation trauma must be objectified by bronchoscopy [14]. The early collection of microbiological wound swabs for differentiated antibiotic therapy during the course of the burn disease has proven its worth [19].

After initial treatment, the patient is admitted to the burn ICU. The acute phase of the disease is usually followed by burn shock. Burn shock is a life-threating condition resulting from the body’s response to the severe burn injury. The management of burn shock involves various medical interventions aimed at stabilizing the patient’s condition and restoring tissue perfusion. Shock therapy in burns primarily involves balanced fluid resuscitation according to hemodynamic monitoring, vasopressor support, pain management, temperature regulation, oxygen therapy, and comprehensive supportive care. There is currently growing concern in burn care regarding increased morbidity and mortality due to fluid over-resuscitation [20]. Fluid resuscitation should be made with the least amount of fluid in order to provide adequate organ perfusion and at the same time preventing consequences of fluid overloading, such as respiratory failure. Crystalloid solutions should be used for fluid resuscitation [14]. There is a recommendation of the ABA to use of human albumin solution in combination with crystalloids to lower resuscitation volumes and improve urine output, especially for patients with larger burns [21]. Fluid resuscitation should be dynamically observed using PiCCO monitoring and correlated by means of echocardiography and clinical assessments [22]. The use of high-dose ascorbic acid (vitamin C) in severely burned patients with more than 20 % TBSA may be considered in the acute phase of burn shock, as vitamin C administration showed limited fluid resuscitation and edema formation [23]. However, there is a lack of controlled randomized studies proving the value of vitamin C in burn shock therapy [24]. Due to a lack of randomized controlled studies, there is no recommendation from the ABA regarding vasopressor therapy [21]. If sufficient organ perfusion and circulation is not achieved with the above-mentioned fluid resuscitation measures, catecholamine therapy with noradrenaline should be given [25]. Dobutamine should be considered for low cardiac output and normotensive blood pressure values [25]. There is no general indication for mechanical ventilation in the case of severe burns. Ventilation should be lung-protective and the earliest possible weaning should be attempted [9]. If prolonged weaning is to be expected, a tracheostomy should be considered at an early stage.

Sedation and pain management should be based on a multimodal approach consisting of analgesics, adjuvants, and nonpharmacological procedures [26]. There is no preference for specific analgesics or combination therapies [26]. Opioids are the foundation of pain management for severe burns [26]. Early mobilization within the first 14 days in the ICU showed a reduction in delirium and slowed muscle atrophy in critically ill adult burn patients [27]. Close cooperation with physiotherapy, occupational therapy, speech therapy, and respiratory therapists is, therefore, required in order to achieve early mobilization of the burn victim. First studies using virtual reality showed a clinical impact on pain, opioid use, and level of anxiety among burn patients in the ICU setting [28]. Therefore, further research should be conducted to see if virtual reality should become a standard of care in burn units. As patients with previous psychiatric disorders have a lower outcome and prolonged ICU stay, psychiatry and psychosomatic medicine should be involved at an early stage of the treatment [29].

It is known that severely burned patients have an acquired factor XIII deficiency, about which little has been described in the literature [30]. However, studies have shown that supplementation of factor XIII could lead to improved hemostasis, wound healing, and general outcome while reducing exposure rates of blood transfusions [30]. Prophylactic anti-infective therapy should not be used [31]. Regular and routine collection of microbiological samples is recommended in order to carry out targeted anti-infective therapy in the event of a clinically relevant wound infection or sepsis [19]. Burn shock can lead multiorgan failure. There are no standardized recommendations regarding the use of organ replacement therapies. The ABA and the German guideline do not give a recommendation regarding early renal replacement therapy (RRT) [21]. To date despite the potential benefits, the optimal timing for RRT initiation remains unclear and further studies are needed [32]. In individual cases, plasma exchange is performed as part of studies to stabilize unstable patients in burn shock by removing the triggering cytokines from the plasma [33]. In the event of lung failure with acute respiratory distress syndrome (ARDS), extracorporeal life support (ECMO) therapy may be considered. Although there is growing evidence and experience using ECMO in burn patients, a careful risk–benefit assessment of the outcome should be considered [34].

Family members should be involved at an early stage as well to clarify the legal care situation and determine the patient’s presumed wishes. In complex decision-making situations, an ethics committee can provide advice and palliative care concepts should be applied accordingly [35]. After surviving the initial burn shock, further phases of shock due to sepsis, wound infections, or pneumonia often occur in the further course of treatment [36]. An early enteral nutrition should be aimed for. If this is not possible orally, feeding tubes or supportive parenteral nutrition should be used [37]. Vitamins and trace elements should be supplemented [37]. In addition, it was shown for the postshock phase that the anabolic synthetic steroid hormone oxandrolone has a positive influence on mortality and length of hospital stay due to the accompanying hypermetabolism in patients with an ABSI greater than 10 [38]. There is also a consensus on medication with propranolol after burn injury by modulating metabolic pathways to achieve improved outcomes [39].

### Surgical treatment concepts

The surgical treatment of burns encompasses various interventions aimed at promoting wound healing, preventing complications, and restoring function as well as aesthetics. The patient-specific treatment approaches involve plastic surgeons, anesthesiologists, nurses specialized in burn care, and other healthcare professionals. Prior any surgical intervention, a thorough assessment of the burn injury is conducted to determine its severity, depth, and extent. Prior surgery, stabilization of the patient needs to be performed regarding airway management, shock therapy along with fluid resuscitation and sepsis therapy.

### Optimal time and method of surgical debridement

Surgical debridement is performed to remove burned, damaged, or infected tissue from the burn wound. This process helps promote wound healing, reduces the risk of infection, and prepares the wound bed for subsequent treatments such as skin grafting. Debridement may be performed surgically with tangential or epifascial debridement or through other methods such as enzymatic or mechanical debridement. Since the approval of a bromelain-based proteolytic enzyme (NexoBrid^®^, MediWound Ltd, Yavne, Israel) in 2012 in Europe, enzymatic debridement has gained in importance in the treatment of 2b–3° burns [40]. A systematic meta-analysis of the current study findings showed that NexoBrid^®^ is a safe, fast, and selective way for eschar removal and showed reduced numbers of operations, length of stay, and cases of wound-associated sepsis [40].

There is an ongoing expert debate about when surgical debridement should be performed in burn patients. A recent German register study was able to show that fewer than half of burn patients underwent early excision and that the TBSA remained the main driver of early excision [41]. Furthermore, the study could show that early excision did not reduce mortality but shortened the inpatient treatment with the time point of admission being a predictive value for early or late excision [41].

An all-encompassing and patient-specific defect coverage concept with rescue options should be considered before any debridement is performed. Skin grafting techniques are the most common surgical procedures used to cover large burn wounds. The various skin grafting techniques and other treatment concepts are presented in detail in the following. In addition to the established surgical procedures, there are numerous research approaches involving skin tissue engineering, 3D bioprinting, and immunoengineering of allogenic skin grafts with the aim of bringing further innovative therapy concepts to the market in the short to medium term [1].

Up to a third of burn victims suffer from scar contractures, which can lead to considerable functional and aesthetic impairments [42]. Of course, the primary aim is to prevent the occurrence of these, but this is not always successful, so secondary corrective plastic surgery procedures are necessary if the scars are stable.

### Autologous skin grafts

Autologous skin grafting has been practiced by European surgeons for more than 200 years and can be considered as standardized therapeutic procedure in the treatment of burn patients [1]. Developments, experience, and knowledge in the field of autologous skin grafting steadily increased, so that it was able to establish itself as the gold standard [1]. Skin grafts provide a temporary or permanent wound covering, promote wound healing, and improve functional and cosmetic outcomes. For large burn areas, the autologous split-thickness skin grafting reaches its limits due to the lack of donor sites and increased donor morbidity [43]. Due to a lower contractility and optimized aesthetic results, full-thickness skin grafts are also used in isolated localizations, e.g., the face and hands [44].

### Allogeneic skin grafts

Allogeneic skin grafts provide temporary wound coverage for extensive burns when sufficient donor sites are not immediately available for autologous skin grafting. On one hand, they protect the wound from infection and prepare it for subsequent autologous skin grafting [45]. On the other hand, rejection reactions due to an immunological reaction against the graft, as well as infections and increased scarring, have been shown to be disadvantageous in application [46]. Skin allografts are currently commercially available as cryopreserved or glycerol-preserved transplants via biobanks or skin tissue banks worldwide. However, no nationwide supply network has yet been established in Germany [47]. A few national skin banks, such as Hannover Skin Bank, are currently focusing on the production of vital allografts from tissue donations following postbariatric surgery, with promising initial results in clinical application [48]. Although the immunogenicity of skin allografts can be reduced by various procedures, such as glycerol preservation, it promotes the destruction of cellular epidermal and dermal components of the skin allografts [49]. Since severely burned patients are usually immunocompromised in the context of burn shock, skin allografts often heal temporarily, but are secondarily rejected after reconstitution of the immune system [49].

### Xenogeneic skin grafts

In addition to the proven method of autologous skin grafting, xenogeneic skin grafts, such as porcine and fish skin, are used temporarily for large-area burns [46]. In most cases, xenogeneic skin grafts are used in a similar way to allogeneic skin grafts for bridging until definitive defect coverage by means of autologous skin grafting. In recent studies, the skin of *Nile tilapia* (*Orechromis niloticus*) has been used in a randomized controlled phase II study to achieve complete re-epithelialization of burn wounds without autologous split-thickness skin grafting [50]. Although the clinical treatment approaches of xenografts have so far shown some promising perspectives, further research projects are required to evaluate the potential in comparison to autologous skin grafting.

### Biological and synthetic skin substitutes

For the treatment of burn wounds, there is currently a plethora of biological and synthetic skin substitutes available on the European and international market [51]. A distinction can be made between epidermal and dermal skin substitutes. Some of the most established skin substitutes are presented in the following. Due to clinically convincing results, SUPRATHEL^®^ (PolyMedics Innovations, Denkendorf, Germany) is considered an established epidermal skin substitute, especially for the treatment of superficial 2a degree burns and scalds. In addition to excellent wound healing results, it also leads to a reduction in costs and mortality [52].

Dermal skin substitutes can be broadly categorized into decellularized dermis constructs of human or animal origin, artificial scaffolds made from various biomaterials or completely synthetic polymers [53]. However, a cross-product disadvantage is the mandatory need to combine a dermal skin substitute with an autologous skin graft to enable complete epidermal and dermal skin replacement. NovoSorb^®^ Biodegradable Temporizing Matrix (BTM) (PolyNovo, Carlsbad, CA, USA) is a fully synthetic dermal skin substitute made of biodegradable polyurethane foam with a temporary nondegradable polyurethane sealant [54]. Numerous studies with BTM have been conducted to investigate the safety and ability for permanent wound closure in combination with autologous split-thickness skin grafts in a two-stage surgical procedure in sheep, pigs, and humans [55]. However, a disadvantage was found to be a period of up to 3 weeks for integration and vascularization of BTM and the resulting prolonged hospitalization times and increased treatment costs [56].

MatriDerm^®^ (MedSkin Solutions Dr. Suwelack AG, Billerbeck, Germany), a dermal, cell-free skin substitute for deep burn wounds, has been commercially available in Germany for several years. The matrix consists of bovine collagen type I, collagen type III, collagen type V, and elastin. As shown in extensive animal studies, MatriDerm^®^ is converted into an autologous matrix of the recipient organism over a period of weeks [57]. MatriDerm^®^ is available in different thicknesses, and a single-stage defect coverage with simultaneous autologous split-thickness skin grafting is clinically considered a significant advantage [57]. In addition, better scar quality and reduced wound contraction were observed [58].

Integra^®^ (Integra Lifesciences, Princeton, NJ, USA) is a dermal skin substitute that consists of bovine collagen type I and shark chondroitin-6-sulfate covered with an outward-facing silicone membrane [59]. The matrix is integrated into the recipient organism and vascularized over a period of approximately 3 weeks before a definitive defect coverage with autologous split-thickness skin grafting can take place after removal of the silicone membrane [59]. Using Integra^®^, an improved scar quality, objectively higher viscoelasticity, and a shorter hospitalization time were described. The prolonged time to integration and vascularization has proven to be a disadvantage [59].

### Autologous cultured keratinocytes and skin grafts

In addition to autologous skin grafts, autologous keratinocytes are also commercially available as spray suspensions or sheets. Spray suspensions (RECELL^®^; Avita Medical Inc., Valencia, CA, USA) or cell sheets (Epicel^®^; Vericel Corporation, Cambridge, MA, USA) from various national and international manufacturers are commercially available and used clinically in the treatment of selected patient cases [60, 61]. Clinical trials for cultured autologous dermo-epidermal skin substitutes for the treatment of severe burns are currently ongoing, and the results to date are promising [62]. However, the use of cultured autografts is limited due to high production costs and a cultivation time of several weeks.

### Flap surgery and microsurgical reconstruction

In cases of deep burns or wounds involving functional structures like tendons, muscles, or bones, flap surgery may be necessary. Skin grafting as well as locoregional flaps can be considered as workhorses, but microsurgical free tissue transfer should be considered on a patient individual basis and must be individualized to the wound bed and surgical needs [63].

### Wound treatment concepts

Surgical management of burns also involves the application of various topical therapies to promote wound healing, reduce pain, and prevent infection. These therapies may include antimicrobial dressings, skin substitutes, growth factors, or specialized wound care products [53]. Due to the variety of available wound dressings and topically applicable substances, these will not be discussed in detail here. To improve wound-drying and re-epithelization, water-filtrated infrared-A irradiation (wIRA irradiation) can be used in clinical routine [64]. In the event of colonization of the burn wound with multiresistant pathogens, the application of specific phage strains for focused anti-infective therapy has been successfully described in isolated cases [65]. The initial results on the use of atmospheric cold plasma or platelet-rich plasma (PRP) in the treatment of burn wounds have shown promising results [66, 67]. However, further studies are required to conclusively assess the value of these treatment methods.

#### Corrective plastic surgery procedures

If scar contractures are present, particularly near joints, these can significantly restrict function in daily life as well as causing aesthetic impairments. One main aim here is to improve the range of motion including the function so that everyday activities can be carried out more easily again. The selection of the required plastic-reconstructive procedure is patient-specific. The localization of the scar contracture as well as its dimensions and influence should be taken into account. An individualized treatment concept can be designed depending on these factors [68].

Raborn and Janis mentioned flaps with a wide variety of options are most frequently used, with skin grafts being the second most common. A combination of techniques (split-thickness grafts and flaps) is often necessary [68]. Furthermore, fat grafting could counteract and prevent skin tethering as well as fill volume defects. For mild to moderate contractures in regions like the axilla or neck (extended), z-plasty or advancement flaps are commonly used. In the case of more extensive expansion and if surrounding tissue is affected, further flap plasty or skin grafts may also be indicated. Nonsurgical options include the use of lasers, local injection of steroids, or a combination of these methods.

### Rehabilitation and post-treatment phase

Rehabilitation plays a crucial role in the postoperative management of burns, aiming to optimize physical, psychological, and social recovery following burn injury. Burn rehabilitation is an ongoing process that continues beyond the acute phase of injury. After treatment in an ICU, the burn patient is usually transferred to a normal ward before being discharged for further aftercare or rehabilitation. In Germany, highly specialized clinics with standardized treatment concepts are available for rehabilitation [69]. Rehabilitation involves a multimodal interdisciplinary approach, similar to the acute phase, and consists of physiotherapy, occupational therapy, and psychological care to support emotional and physical recovery. Prerequisites for the start of rehabilitation are almost healed epithelial defects, no need for further surgical intervention and a cardiorespiratory stable patient who is already partially mobilized. There are different types of rehabilitation that address different goals.

Primary rehabilitation takes place immediately after inpatient treatment in the acute hospital and focuses on scar and wound healing. The aim of postacute rehabilitation is to learn and regain independence in everyday life. The time frame comprises approximately 6–12 weeks. Follow-up rehabilitation is aimed for burn-injured patients who have already regained most of their independence in everyday life. The focus here is on improving function and fitness as well as mental health care. This type of rehabilitation is expected to require 3–8 weeks. Inpatient treatment is indicated if outpatient therapy is unsatisfactory or if a deficit persists.

The multimodal therapy strategies of rehabilitation include scar therapy, movement therapy, contracture treatment, psychological support for the patient and family members, pain therapy, treatment of concomitant illnesses and consequential damage including amputations, as well as (social) reintegration.

Scar tissue can cause functional and aesthetics concerns. Therefore, scar management techniques such as scar massage, pressure garments, silicone sheets, laser therapy, or surgical scar revision may be employed to optimize functional and cosmetic outcomes and improve the patient’s quality of life [70]. Due to the disfiguring nature of the scarring on visible areas, those affected often experience psychosocial impairments and feel socially stigmatized [71]. Surgical scar revision may be performed using Z-plasty technique, autologous skin grafts, or percutaneous collagen induction [70]. In addition to scar therapy, the focus is also on lifelong skin care of the transplanted areas and split-thickness skin harvesting sites with moisturizing external agents. Strict sun avoidance of the affected areas should also be adhered to in order to prevent secondary malignancies and pigmentation disorders.

Long-term follow-up care ensures that patients receive ongoing support, monitoring, and interventions to address evolving needs, improve quality of life, and facilitate successful reintegration into society for burn survivors. Changes in immune function may be associated with higher morbidity in burn injury survivors. A number of secondary pathologies such as increased risk of cancer, cardiovascular disease, nervous system disorders, diabetes, musculoskeletal, gastrointestinal, and psychological disorders are associated with long-term survivors of burn injuries [72]. Patients with ICU stays have an increased risk of suffering from acute as well as long-term mental disorders [73]. Post-traumatic stress disorders often occur in patients with severe burns [74]. Reintegration into society can cause increased psychological stress [73]. Adults who have suffered burns in childhood have an increased risk of mental and physical illness. Constant re-evaluation is also indispensable during aftercare. Overall burn survivors have an increased rate of suicidal ideation. However, the data regarding suicidality in survivors are limited, but they often have various risk factors that are associated with an increased risk of suicidality in the general population [75]. Even long after the acute phase and in an advanced rehabilitation status, occupational therapists are essential for supporting the recovery of social as well as professional participation. However, resources are limited [76]. Many survivors first realize during the rehabilitation process that recovery is a progressive and a lifelong process. However, due to a lack of understanding about wound care and rehabilitation progress, survivors are confronted with difficulties in reintegration [72]. Generally, environmental and personal factors influence participation in employment and work performance.

For patients with limb loss or functional impairment, prosthetic devices and orthotic interventions may be prescribed to enhance mobility, functionality, and independence [77]. Bionic prostheses are also used in isolated cases. In burn survivors, nociceptive, neuropathic, and mixed pain can be debilitating during rehabilitation and afterward. Efficient pain therapy in accordance with the WHO scheme is, therefore, crucial. Additive procedures based on a multimodal treatment concept are necessary to reduce an increased risk of pain chronification.

## Conclusions

Due to the complexity of the treatment, severely burned patients are treated in highly specialized burn units. Comprehensive knowledge of the various components and players involved in the care of severely burned patients is mandatory for all surgeon to achieve the best possible outcome for patients. Germany currently offers a high level of nationwide burn care. There are already reliable studies on many therapeutic procedures. However, there is still a need to substantiate some treatment concepts with further studies. In addition, recent developments in the field of technological progress and new skin replacement materials must be critically evaluated in the coming years with regard to their clinical applicability in burn patients.
